# A new approach towards volumetric assessment of left ventricular function with MSCT

**DOI:** 10.2349/biij.2.3.e50

**Published:** 2006-07-01

**Authors:** S Yamamoto, S Hamada, M Miyamoto, J Masumoto, M Komizu, G Iinuma, N Moriyama

**Affiliations:** 1 Research Center for Cancer Prevention and Screening, Tokyo, Japan; 2 Department of Radiology, Osaka University Graduate School of Medicine, Osaka, Japan; 3 Cuscreed Co. Ltd., Tokyo, Japan

**Keywords:** ECG-gating, 4D imaging, LV volume, ejection fraction

## Abstract

Cardiovascular CT is considered the diagnostic standard for establishing the presence of a functional and dynamic imaging system. It is difficult, however, to estimate the ventricular motion and volumes that are processed using hundreds and thousands of CT images, in a few moments.

The main concept and design of our work are two fold — the development of effective semi-automatic tools for measuring the sequential left ventricular volumes from the hundreds or thousands of cardiac trans-axial images, and providing a simple interface with an interactive diagnostic tool for the volumetry of left ventricle and valuable cardiac 4D visualisation.

We converted ten and more sequential volume data sets of the heart acquired from retrospective ECG-gating helical scan into 3D images by volume rendering. These sequential 3D images could be displayed as a movie (4D cardiac image) file. Furthermore, we developed a method for semi-automatic calculation of ejection fraction (EF) and cardiac cycle (%)-volume (ml) curve for estimation of the motion and the volume of the left ventricle. This method involved the use an interactive selection tool in the region of interest (ROI). All 3D processing methods, such as, cutting objects, segmentation, and image fusion were based on mask processing data. We now describe the software developed for cardiac 4D imaging and the estimation of ventricular volume.

## INTRODUCTION

Multislice computed tomography (MSCT) offers good cardiac image quality using ECG synchronised technique, producing no motion artefacts [1-5]. The heart can be visualised by static or dynamic means. Static visualisation is used to obtain morphological information, while dynamic imaging permits functional evaluation. Some reports have described the accurate estimation of ventricular volume and motion with MSCT [6–8]. It is difficult, however, to estimate the ventricular motion and volumes on the CT apparatus itself because of the large cardiac volume data required for a high performance image processing. For reconstructing the time-series images from the ECG signal, hundreds or thousands of trans-axial images are produced, and the interactive reading tool for a cardiac CT examination is expected to use these for efficient diagnosis.

As the development of computer hardware and software has been incredibly fast and inexpensive, a system of real-time visualisation and image analysis should provide the user with good quality image and movie display. First, we developed effective semi-automatic tools for measuring the sequential left ventricular volumes from the hundreds or thousands of cardiac trans-axial images. We adopted this technique and made several improvements to overcome the problem of automatic calculation of the left ventricular volumes for different motion phases acquired from the retrospective ECG-gating helical scan. Second, we extended this technique to display 3- and 4D images of the heart with real-time volume rendering on the personal computer (PC).

By this, we reduced the time and the effort taken for cardiac function analysis and 4D display. We introduced a design-integrated system for cardiac data acquisition and various tools for cardiac function analysis.

## RETROSPECTIVE ECG-GATING

**Figure 1:**
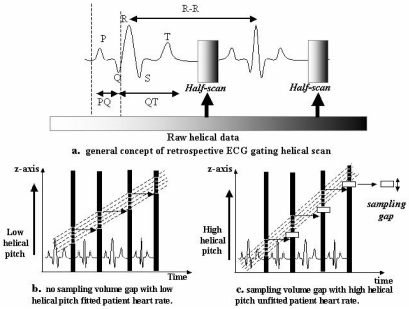
Reconstruction procedure with retrospective ECG-gating (four slices). (a) Retrospective ECG-gating is able to overcome the limitation of prospective ECG-triggering with inconsistent heart phase scanning when arrhythmia is present. Arbitrary phases of the heart motion were reconstructed from the continuous raw helical data; (b) Slow table motion during helical scanning and simultaneous acquisition of four slices and the ECG trace are employed to perform an oversampling of scan projections; (c) Higher helical pitch sets the appropriate sampling gap of the raw data space due to misalignments between helical pitch and heartbeat.

Retrospective gating is needed for helical scanning, and ECG gating has been used for years in magnetic resonance imaging (MRI). It consists of contiguous data acquisition with parallel registration of the patient’s ECG (Figure 1). Subsequent segmentation of the acquired data enables re-ordering and re-grouping of certain data in concord with the registered ECG, for clear visualisation of defined time points in cardiac cycle (Figure 1a). In this approach, slow table motion during spiral scanning and simultaneous acquisition of four slices and the digital ECG trace are employed to perform an over sampling of scan projections (Figure 1b). The sampling gaps in the raw helical data with ECG-gating were generated asynchronously between helical pitch (higher helical pitch) and heart rate (Figure 1c). This retrospective synchronisation enables the visualisation of the myocardium of the same region during diastole and systole by using the same data set. The application of this method in CT opens a multitude of possibilities.

The advantages of ECG gating over ECG triggering is that the data acquisition is independent of the patient’s heart rate, and it is possible to visualise the imaged region during different times in the cardiac cycle. This permits functional evaluation. The disadvantage is the prolonged data post processing procedure and the associated higher radiation dose.

## MATERIALS AND METHODS

### Network System for Cardiac Data Acquisition from Multi-slice CT

We installed 4D cardiac analysis software on a standard PC with Pentium 4 3.00 GHz CPU, and 2.00 GB RAM on Microsoft Windows XP and Intel Core Duo, 2.00 GHz CPU and 2.00 GB RAM on Mac OS X. The processing and the image rendering tools of the software are based on the open-source libraries with OsiriX [9]. Cuscreed Co. Ltd. (Tokyo, Japan) did the optional development for the algorithm of cardiac cycle (%) – LV volume (ml). The software can be connected to any DICOM 3.0 model database (patient, study, series, image) via an Ethernet TCP/IP network (100-Mbps Fast Ethernet). The users can transfer the DICOM images from MSCT (Aquillion VZ: Toshiba, Nasu, Japan) directly to the PC, with the cardiac analysis software. ECG monitoring systems (Dyna Scope, Model: DS-2151, Fukuda Denshi, Tokyo, Japan) were implemented on the MSCT. The users can also export images, ECG-signals, and movies in PC standard format for local storage, presentation, and print.

### Technical Procedure for Cardiac 4D Imaging

**Figure 2:**
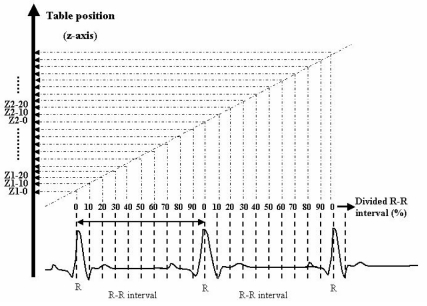
Reconstruction procedure for 4D cardiac imaging using ECG-gating technique. ECG-gating permits visualisation of multiple time points during the cardiac cycle out of one helical CT data set. For the first end-diastolic phase, the image acquisition window was centred on the peak of the R wave. For the second data set, the centre was positioned at 10 % of the R–R interval, and for subsequent data sets, the centre of the image acquisition window was moved toward the end of the cardiac cycle in increments of 10%. The groups of slices Z1-10 and Z2-10 or Z1-20 and Z2-20 in the figure were reconstructed from the same phase of R–R interval. Each volume set was converted to a 3D image, and these sequential 3D images were displayed as 4D cardiac images.

ECG leads were placed on both wrists and the left forearm of the patients to avoid artefacts. Following a scout view for positioning, the contrast-enhanced CT data were acquired during a single breath-hold. Patients inhaled 3.0 l/min of oxygen prior to the acquisition, while the scan parameters were established. Fifty seconds after intravehicular injection of the contrast medium (100 ml of 320 mg I/ml at 1.2 ml/sec: Iohexol 320, Daiichi Pharmaceutical Co., Ltd., Tokyo, Japan), the scan was started with the ECG-gating technique. The exact start and end points of the helical scanning synchronised ECG record were monitored on the multislice CT console. The parameters used were 0.5 sec per rotation, and the value for 2-mm-detector row collimation of the helical pitch (length of table translation divided by X-ray beam width) was 0.2:1. The time of breath hold was about 75 rotations through 12 cm during 37.5 sec. The X-ray tube voltage and current were 140 kV and 160 mA, respectively. The volume data sets for phases of cardiac motion were prepared as 10 sets reconstructed for every 1mm interval using 50% overlap reconstruction from raw-helical CT data. For each patient, 10 sets of volume data were created by using both half-scan and segment reconstruction based on the retrospective ECG-gating helical scanning [2]. For the first end-diastolic phase, the image acquisition window was centred on the peak of the R wave. For the second data set, the centre was positioned at 10% of the R-R interval, and for subsequent data sets, the centre of the image acquisition window was moved toward the end of the cardiac cycle in increments of 10% (Figure 2). The software can obtain these 10 volume data sets as a DICOM series and can take in as many as the memory size of the PC hardware enables.

## IMAGE PROCESSING AND ANALYSIS IN CARDIAC CT EXAMINATION

### 4D Cardiac Imaging

After loading the 10 sets of volumes for all cardiac trans-axial slices, the user can easily change the order of the series to make sequential 4D cardiac image.

**Figure 3:**
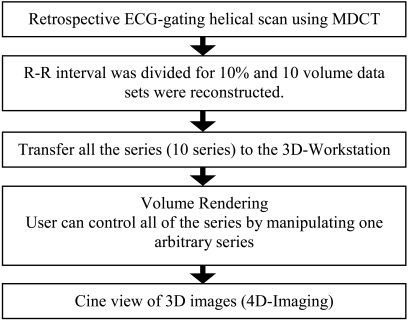
Flowchart of cardiac 4D display. All series produced by the ECG-gating technique were transferred to the 3D workstation. The 3D-Workstation can load the entire series and sort it as sequential data. Users can control the entire series by manipulating just one arbitrary series. The remaining nine sets of series were processed in sync with each other by manipulating one series data.

To reduce time and effort, the parameters of volume rendering (transfer function of colour and opacity curve, cutting, panning, zooming, and others) were automatically synchronised with just one volume operation. The remaining nine sets of series were processed in sync with a series of interest. Figure 3 shows the flowchart of 4D cardiac imaging.

### Ejection Fraction

The EF, a parameter of global ventricular function, is calculated from the volumes at end diastolic EDV and end systolic ESV as:

$$EF=\frac{EDV-ESV}{EDV}\times 100\%$$

This is the single most powerful prognostic indicator, and its value correlates with the risk of morbidity and mortality in different types and stages of heart disease. The presence of an abnormality in the regional wall motion suggests ischemic heart disease, the location of the hypokinesis correlates loosely with the coronary artery that is affected, and the size of the functional defect suggests the amount of myocardium that is ischemic or infracted. In our software, semi-automatic EF calculation was applied with simple segmentation and volume calculation using voxel counting to avoid the error of segmentation at the level of tricuspid valve.

**Figure 4:**
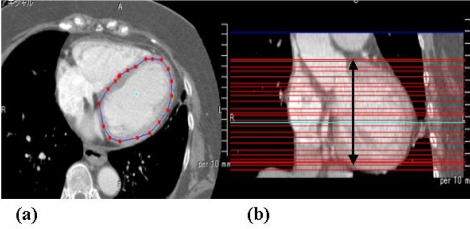
Segmentation for selection of the left ventricle. (a) The ROI were manually placed on the left vetricle with end-diastoric phase. Users can manipulate the ROI with deformation, panning, and zooming; (b) Selected ROI on the skipped slice reveals that the slice was interpreted and cut along the Z axis.

**Figure 5:**
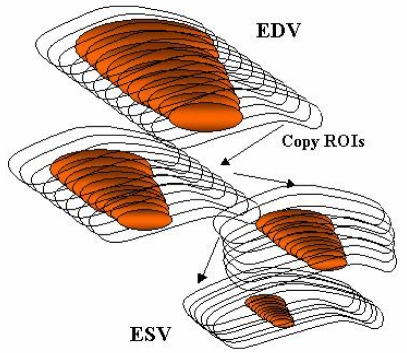
An illustration of the method involving copying the volume of interest (VOI) from EDV volume dataset to ESV volume dataset. The ROI on the slice of end-diastolic phase were copied to the same level of slice for other phases.

The procedure to calculate the semi-automated EF is as follows:

1. Selection of the arbitrary ROI around the left ventricular cavity on trans-axial images in end-diastolic phase. (The ROIs were interpolated between all slices within the selected area). Figure 4 shows the scene of arbitrary selection of left ventricular region for several slices. Irregular gaps among the slices were converted to regular intervals by automatic interpolation.
2. Copying the ROIs of the end-diastolic phase to all other phases. Left ventricular cavities with all phases covered by left ventricular cavity at end-diastolic phase. (Figure 5)
3. Estimation of threshold Hounsfield Unit (HU) value for segmentation of left ventricular cavity using histogram analysis applied Mode method [10,11]. The left ventricular cavity filled with contrast media was segmented by statistical method (mode value) depending on the HU value histogram. Threshold HU values were decided as a midpoint value between two mode values on a bimodal histogram drawing around the ROI of the left ventricular cavity.
4. Automatic drawing of the cardiac cycle (%) – volume (ml) curve and EF calculation.

## RESULTS AND DISCUSSION

**Figure 6:**
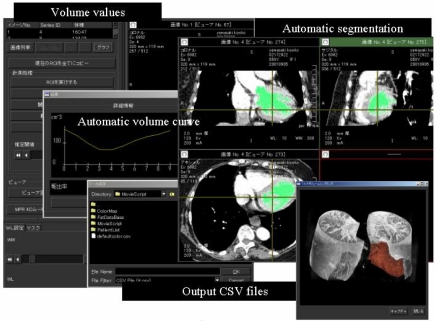
Multi-display of the results of cardiac function analysis. Calculated phase (%)–volume curve is shown in the figure. The user can easily fix the segmented area of left ventricle and re-calculate the volumes.

**Figure 7:**
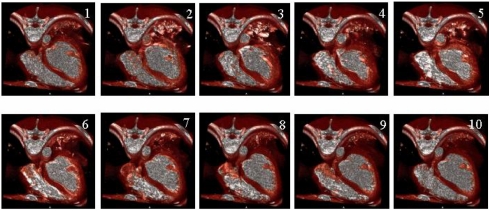
Resultant display of 4D left ventricular imaging. R–R intervals were divided into ten phases using retrospective ECG-gating.

The resulting 3D image set consists of 109 slices per temporal frame and 10 temporal 3D frames per cardiac cycle. The results of the segmentation of LV volume and cardiac cycle (%) – volume (ml) curve are displayed in Figure 6. EF can be calculated from the cardiac cycle (%) – volume (ml) curve. A 4D view of the cardiac motion was reconstructed using the fast volume rendering technique for each cardiac cycle (Figure 7 and Movie1). Modern helical CT systems offer a rotation time of 0.4-0.5 sec. With this technique, in combination with ECG-gating, diastolic images almost free of motion artefacts can be acquired from patients with a moderate heart rate.

**Figure 8:**
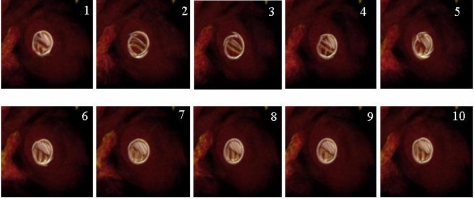
4D view of the prosthetic valve. The operator can easily check the state of the closing valve motion following prosthetic valve replacement.

One of the most effective techniques for cardiosurgery is temporal 3D display of prosthetic valve after prosthetic valve replacement (Figure 8 and Movie 2). The operator could easily check the state of the closing valve motion in this view. What was once a time-consuming task is improved by semi-automatic processing of the multi-volume dataset. Most high-performance 3D workstations offer only a simple measurement tool (e.g., plot profile, histogram, and filters) applied 2D or one volume dataset. The examples presented in this article, however, do not represent a completed project. Need exists for a comprehensive evaluation procedure covering cardiac diseases, such as, coronary artery disease, myocardial infarction, cardiac anomaly, and others. Our further research would include the complete development of the software and the analysis using cardiovascular CT system.

## CONCLUSION

The DICOM-supported software for 4D cardiac imaging and function analysis with online connection to multislice CT apparatus is useful to comprehend the left ventricular motion and volumes.
